# CNOT1 cooperates with LMNA to aggravate osteosarcoma tumorigenesis through the Hedgehog signaling pathway

**DOI:** 10.1002/1878-0261.12043

**Published:** 2017-03-06

**Authors:** Dong‐dong Cheng, Jing Li, Shi‐jie Li, Qing‐cheng Yang, Cun‐yi Fan

**Affiliations:** ^1^ Department of Orthopedics Shanghai Jiao Tong University Affiliated Sixth People's Hospital China; ^2^ State Key Laboratory of Oncogenes and Related Genes Shanghai Cancer Institute Renji Hospital Shanghai Jiao Tong University School of Medicine China

**Keywords:** CNOT1, Hedgehog signaling pathway, LMNA, osteosarcoma, proliferation

## Abstract

While treatments for childhood osteosarcoma have improved, the overall survival for this common type of bone cancer has not changed for three decades, and thus, new targets for therapeutic development are needed. To identify tumor‐related proteins in osteosarcoma, we used isobaric tags in a relative and absolute quantitation proteomic approach to analyze the differentially expressed proteins between osteosarcoma cells and human osteoblastic cells. Through clinical screening and functional evaluation, CCR4–NOT transcription complex subunit 1 (CNOT1) correlated with the growth of osteosarcoma cells. To date, the mechanisms and regulatory roles of CNOT1 in tumors, including osteosarcoma, remain largely elusive. Here, we present evidence that knockdown of CNOT1 inhibits the growth of osteosarcoma *in vitro* and *in vivo*. Mechanistically, we observed that CNOT1 interacted with LMNA (lamin A) and functioned as a positive regulator of this intermediate filament protein. The RNA‐seq analysis revealed that CNOT1 depletion inhibited the Hedgehog signaling pathway in osteosarcoma cells. A rescue study showed that the decreased growth of osteosarcoma cells and inhibition of the Hedgehog signaling pathway by CNOT1 depletion were reversed by LMNA overexpression, indicating that the activity of CNOT1 was LMNA dependent. Notably, the CNOT1 expression was significantly associated with tumor recurrence, Enneking stage, and poor survival in patients with osteosarcoma. Examination of clinical samples confirmed that CNOT1 expression positively correlated with LMNA protein expression. Taken together, these results suggest that the CNOT1–LMNA–Hedgehog signaling pathway axis exerts an oncogenic role in osteosarcoma progression, which could be a potential target for gene therapy.

**Abbreviations**
CCK‐8: Cell Counting Kit‐8
CHX: cycloheximide
CNOT1: CCR4–NOT transcription complex subunit 1
Co‐IP: co‐immunoprecipitation
GO: gene ontology
GSEA: Gene Set Enrichment Analysis
IHC: immunohistochemistry
iTRAQ: isobaric tags for relative and absolute quantification
KEGG: Kyoto Encyclopedia of Genes and Genomes
LMNA: lamin A
OS: overall survival
TFS: tumor‐free survival

## 1 Introduction

Osteosarcoma is the most common primary malignant bone tumor in children and adolescents. In the past, amputation was the standard treatment, with a 5‐year survival rate of 20% (Berlanga *et al*., 2016; Coventry and Dahlin, 1957). With the application of neoadjuvant chemotherapy combined with limb salvage surgery, the 5‐year survival rate has risen to 70% (Sampo *et al*., 2008). However, overall survival (OS) has remained unchanged for over three decades despite attempts to improve outcomes via modification of chemotherapy drugs (Yan *et al*., 2016). Currently, many patients are not sensitive to chemotherapy and have a poor prognosis (Yao *et al*., 2012). Therefore, there is an urgent need to discover novel biomarkers and therapeutic targets for the diagnosis and treatment of osteosarcoma.

Quantitative proteomics provides an approach for the rapid identification of new protein targets and helps to elucidate the underlying molecular events associated with the development and progression of cancer (Parker and Borchers, 2014; Ren *et al*., 2010). Quantitative proteomic analysis has been reported in various tumors, including oral squamous cell carcinoma (Jou *et al*., 2014), colon cancer (Li *et al*., 2013), and gastric carcinoma (Subbannayya *et al*., 2015). Currently, osteosarcoma cell lines as osteoblastic cell models have been extensively used to investigate bone biology (Bernardini *et al*., 2014). Previously, a comparative proteomic analysis of plasma membrane proteins was performed between human osteosarcoma cells and osteoblastic cells (Zhang *et al*., 2010). A total of 342 proteins were identified in that study, 68 of which were differentially expressed and one protein CD151 was verified by immunohistochemistry. In addition, proteomic studies have been carried out between osteosarcoma tissues and normal tissues to identify a group of proteins potentially involved in osteosarcoma malignancy (Folio *et al*., 2009; Kubota *et al*., 2013; Li *et al*., 2010).

In the present study, comparative proteomic analysis was performed between osteosarcoma cell lines and an osteoblastic cell line. Through clinical analysis and functional screening, CNOT1, a member of the human CCR4–NOT deadenylase complex, was found to be related to osteosarcoma proliferation. Accumulating evidence suggests that the CCR4–NOT complex is involved in the regulation of mRNA deadenylation, mRNA decay, and cell viability (Ito *et al*., 2011). This study demonstrated that knockdown of CNOT1 inhibited osteosarcoma cell growth *in vitro* and *in vivo*. In addition, CNOT1 was found to interact with lamin A (LMNA) and affect its protein stability in osteosarcoma. Knockdown of CNOT1 could inhibit Hedgehog signaling pathway. Activation of the Hedgehog signaling pathway has been implicated in the development of many human malignancies, including prostate cancer (Xu *et al*., 2014), ovarian cancer (Li *et al*., 2016), and breast cancer (Zhou *et al*., 2016). Overexpression of LMNA could rescue CNOT1 knockdown‐mediated tumor inhibition and Hedgehog signaling pathway inhibition. Notably, we found that CNOT1 was an independent prognostic factor for tumor‐free survival (TFS) and OS in patients with osteosarcoma. A positive correlation between CNOT1 and LMNA expression was detected in osteosarcoma tissues. Taken together, knockdown of CNOT1 inhibited osteosarcoma growth through the Hedgehog signaling pathway via its association with LMNA, suggesting a candidate orientation for osteosarcoma treatment.

## 2 Materials and methods

### 2.1 Cell lines and cell culture

Three osteosarcoma cell lines (MNNG/HOS, MG63, and U2OS) and one human osteoblastic cell line (hFOB 1.19) were used. The cells were maintained at 37 °C in a humidified air atmosphere containing 5% CO_2_. They were cultured in Dulbecco's modified Eagle's medium (MNNG/HOS and MG63) or RPMI‐1640 medium (U2OS) supplemented with 10% fetal bovine serum (Biowest, South America Origin), 100 U·mL^−1^ penicillin (Sigma‐Aldrich, St Louis, MO, USA), and 100 mg·mL^−1^ streptomycin (Sigma‐Aldrich). The hFOB 1.19 cell line was cultured according to ATCC protocols.

### 2.2 Proteomic analysis

**Figure 1:**
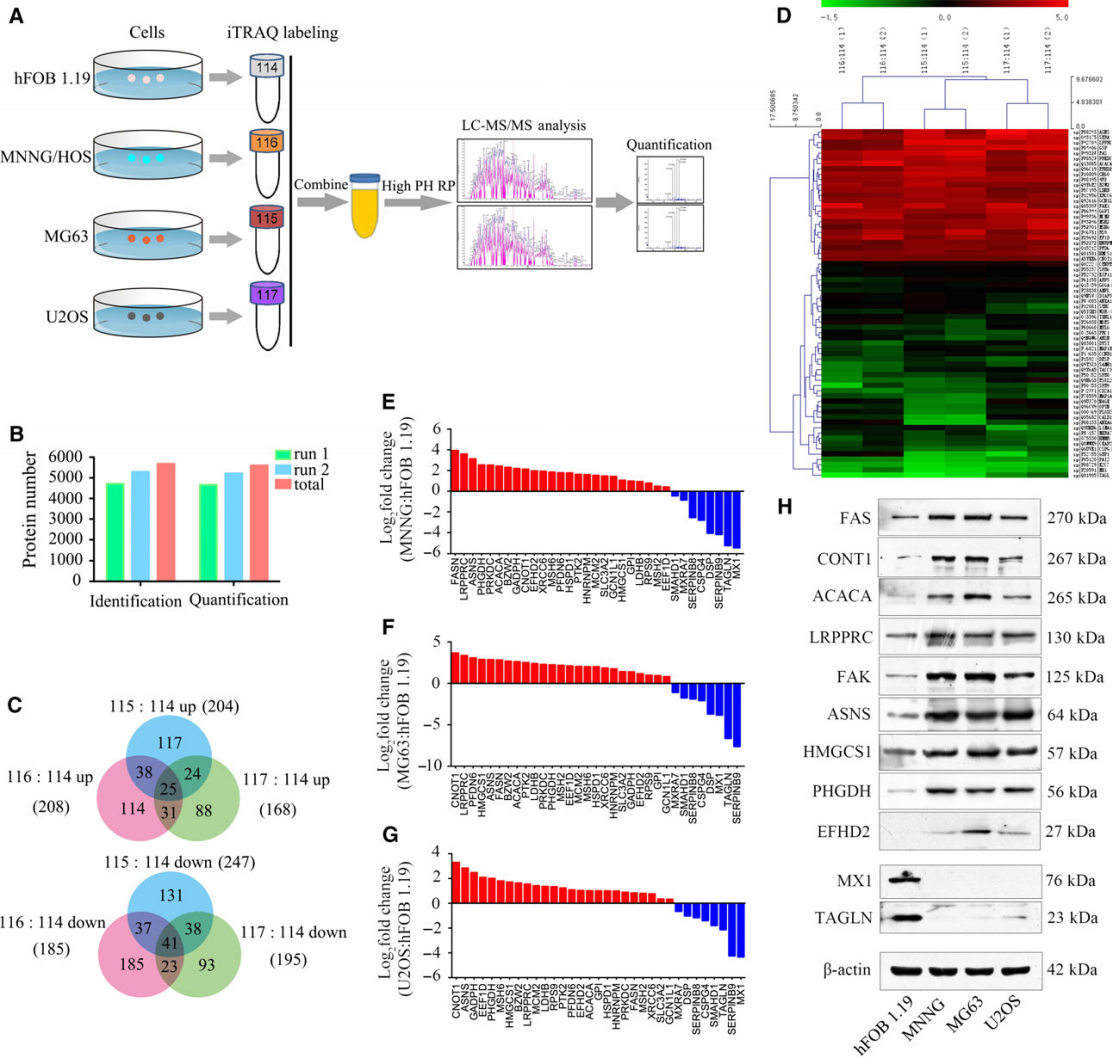
Experimental workflow and results of the quantitative proteomic analysis. (A) The flow chart of the proteomic analysis. (B) The number of identified and quantified proteins in run 1 and run 2. (C) The number of up‐regulated and down‐regulated proteins in osteosarcoma cells. (D) A heat map of dysregulated proteins in osteosarcoma. (E–G) The mRNA expression levels of 25 up‐regulated and eight down‐regulated proteins in osteosarcoma cells compared with hFOB 1.19 cells were verified by qRT‐PCR. (H) Representative blots display the protein expression of nine up‐regulated and two down‐regulated proteins. β‐Actin was used as an internal control.

The cells were cultured to 80% confluence and collected. The whole‐cell protein was extracted. The proteomic analysis was performed as described previously (Lin *et al*., 2013). Briefly, 100 μg of the hFOB 1.19, MNNG/HOS, MG63, or U2OS cell lysate was labeled with iTRAQ labeling reagents 114, 115, 116, or 117 (Applied Biosystems, Foster City, CA, USA). After strong cation exchange and NanoLC‐MS/MS analysis, protein identification and iTRAQ quantitation were performed using proteinpilot4.1 software (AB SCIEX, Toronto, OH, USA). Two physical replicates were performed. Figure 1A shows the flow chart of the proteomic analysis. The differentially expressed proteins underwent gene ontology (GO) analysis and Kyoto Encyclopedia of Genes and Genomes (KEGG) pathway analysis (Lin *et al*., 2013).

### 2.3 RNA isolation and quantitative Real‐time PCR assays

Total RNA from human tissue samples and cultured cells was extracted with TRIzol reagent (Invitrogen, Carlsbad, CA, USA) and quantified with a Nanodrop 2000 spectrophotometer (Thermo Fisher Scientific, Waltham, MA, USA) following the manufacturer's protocol. First‐strand cDNA was synthesized with a PrimeScript RT Reagent Kit (TaKaRa, Shiga, Japan). The cDNA templates were combined with SYBR Green premix with RoxII (Takara, Dalian, Liaoning, China) to perform quantitative PCR reactions. All reactions were performed in a 10‐μL reaction volume in triplicate. The expression level of genes was measured using the comparative Ct method (Yang *et al*., 2016). Information on the primer sequences used is presented in Table S2.

### 2.4 Western blot analysis

Equal amounts of protein samples extracted from the harvested cells were separated using 6% or 8% SDS/PAGE and were then transferred to NC membranes (Millipore, Billerica, MA, USA). After blocking in 5% (w/v) nonfat milk, the membranes were incubated with primary antibodies overnight. Information on the primary antibodies used is presented in Table S3. The secondary antibody was anti‐rabbit IgG (1 : 5000; Sigma‐Aldrich) or anti‐mouse IgG (1 : 5000; Sigma‐Aldrich). Subsequent visualization was performed with SuperSignal West Femto Maximum Sensitivity Substrate (Thermo Fisher Scientific).

### 2.5 Oligonucleotide transfection and stable cell line generation

Predesigned siRNA (Ribobio, Guangzhou, China) and plasmids (Public Protein/Plasmid Library, Nanjing, China) were transfected into cells using Lipofectamine 2000 Reagent (Invitrogen) following the manufacturer's protocol. To construct a stable cell line, scramble control or CNOT1‐specific shRNA particles (Biotend, Shanghai, China) were infected into osteosarcoma cells according to the manufacturer's protocol.

### 2.6 Cell proliferation assays and cell cycle analysis

#### 2.6.1 Cell proliferation

The cell proliferation assay was performed using a Cell Counting Kit‐8 (CCK‐8; Dojindo, Mashikimachi, Japan). Briefly, a mixture of 10 μL of CCK‐8 and 100 μL of DMEM was added to each well followed by incubation for 2 h. The absorbance at 450 nm was then measured. Each measurement was taken in triplicate, and the experiments were repeated twice.

#### 2.6.2 Cell cycle

The cell cycle was analyzed using a Cell Cycle Detection Kit (Kaiji, Nanjing, China) and a FACSCalibur flow cytometer (BD Biosciences, San Jose, CA, USA). The results were analyzed with modfit software (BD Biosciences). The assays were independently conducted three times.

### 2.7 Colony forming assay

Forty‐eight hours after siRNA transfection, 1 × 10^3^ MNNG/HOS, MG63, or U2OS cells were seeded in six‐well plates. After 10 d, cells in each well were fixed with 100% methanol for 30 min and stained with 0.1% crystal violet for 30 min. Finally, the cell colonies were counted. The assays were independently conducted three times.

### 2.8 Migration and invasion assays

Cell migration and invasion assays were performed in a 24‐well plate with 8‐mm pore size chamber inserts (Corning, New York, NY, USA). For the migration assays, 48 h after siRNA transfection, 5 × 10^4^ cells per well were placed in the upper chamber on an uncoated membrane. For the invasion assays, 1 × 10^5^ cells per well were placed in the upper chamber on a Matrigel‐coated membrane. In both assays, cells were diluted with serum‐free culture medium. The lower chambers contained 800 μL of medium with 10% FBS. The cells were incubated for 12 and 16 h for the migration and invasion assays, respectively. Then, the cells that had migrated through the membrane inserts were stained and counted. The assays were independently conducted three times.

### 2.9 Animal experiments

All animal experiments were approved by the Ethics Committee of the Shanghai Jiao Tong University Affiliated Sixth People's Hospital (YS‐2016‐064, 24 February 2016). For tumor growth assays, MNNG/HOS cells stably expressing sh‐control or sh‐CNOT1 were injected subcutaneously into the left scapulas of nude mice (6‐week‐old BALB/c‐nu/nu, 10 per group, 2 × 10^6^ cells per mouse). The tumor volume was monitored twice a week and was calculated using the formula V = 1/2 × length × width^2^. After four weeks, the mice were euthanized. For histological analysis, the primary tumors were harvested at necropsy and fixed in 10% formalin.

### 2.10 Co‐immunoprecipitation (Co‐IP) and mass spectrometry protein identification

Co‐IP was performed in MNNG, MG63, and U2OS cells. Equal amounts of protein (3000 μg) were incubated with antibodies at 4 °C overnight, and the mixture was incubated with protein A/G magnetic beads at 4 °C for 3 h. The beads were washed using phosphate‐buffered saline containing 1‰ Triton X‐100 and eluted using 2× protein loading buffer at 100 °C for 10 min. For silver staining, IgG‐bound or CNOT1‐bound proteins were resolved by SDS/PAGE and stained with a silver staining kit (Beyotime Biotechnology, Shanghai, China). For mass spectrometry analysis, CNOT1‐bound proteins were resolved by SDS/PAGE and stained with Coomassie Blue R250 (Solarbio, Beijing, China). After destaining, reduction, and trypsin digestion for 12 h, the peptides were extracted using acetonitrile. The peptides were analyzed using a NanoLC system (NanoLC‐2D Ultra; Eksigent, Dublin, CA, USA) equipped with a Triple TOF 5600 mass spectrometer (AB SCIEX). Protein identification was performed with proteinpilot4.1 software (AB SCIEX). For this study, a strict unused confidence cutoff > 1.3 and peptides ≥ 2 were used for protein identification.

### 2.11 Confocal immunofluorescence

Confocal immunofluorescence microscopy was performed on MNNG, MG63, and U2OS cells. Briefly, cells were fixed and incubated with rabbit polyclonal anti‐CNOT1 antibody and mouse monoclonal anti‐LMNA antibody at 4 °C overnight and then incubated with secondary antibodies (Invitrogen) for 1 h. Finally, the cells were incubated with DAPI (Sigma‐Aldrich) for 5 min and viewed with a Fluoview FV1000 microscope (Olympus, Tokyo, Japan).

### 2.12 Clinical samples and immunohistochemistry (IHC)

Twenty human tissue sample pairs in which each pair consisted of an osteosarcoma sample and a corresponding nontumor tissue sample were obtained at Shanghai Sixth People's Hospital. Total RNA was extracted for clinical screening. The tissue microarray contains a total of 151 patients who had been diagnosed with osteosarcoma at the Shanghai Sixth People's Hospital. All osteosarcoma samples were confirmed by postoperative pathology. They received primary surgical treatment and preoperative and postoperative neoadjuvant therapy. The median age of the patients was 20 years old (range: 6–84 years). The follow‐up period ranged from 24 to 68 months, and the median time was 40 months. Ethics approval was obtained from the local hospital ethic committees, and written informed consent was obtained from each patient before sample collection. A standard IHC staining procedure was followed. Paraffin‐embedded sections were cut at a 4 μm thickness and then dewaxed in xylene and treated with microwave heating at 60 °C for 20 min in an EDTA buffer (pH 9.0) for antigen retrieval. Each slide was blocked for endogenous peroxidase activity by incubation in 0.3% H_2_O_2_ for 10 min and then incubated at 37 °C with a 1 : 100 dilution of primary antibodies against CNOT1 (Proteintech, Wuhan, China), LMNA (1 : 200; Abcam, Cambridge, MA, USA), or Ki‐67 (1 : 200; Dako, Glostrup, Denmark). Slides were then rinsed three times in PBS, incubated for 30 min with an EnVision staining kit (Dako), washed three times in PBS, and stained for 3–10 min in a moist chamber at room temperature using DAB. Slides were counterstained with hematoxylin and dehydrated in a graded ethyl alcohol series (70%, 90%, 100%). The primary antibody was substituted with PBS in sections used as negative controls. Assessment of IHC staining was made independently by two expert pathologists. Any discordance was solved through consensus discussion. The IHC signal intensities were scored as negative, weak, positive, or strong. For Ki‐67, the percentage of Ki‐67‐positive cells was calculated.

### 2.13 Statistical evaluation

The data were compiled and analyzed using spss version 21.0 (Chicago, IL, USA). Comparisons between different groups were made using chi‐square tests. The independent prognostic significance of the parameters was estimated using the Cox proportional hazards model. The correlation analysis between CNOT1 and LMNA was performed by the Spearman correlation test. Survival curves were generated by the Kaplan–Meier method (Bell *et al*., 2016). The TFS time is the period from surgery to the presence of new local lesions. The OS time was defined as the length of time between the surgery and death. A significant result was considered at *P* < 0.05.

## 3 Results

### 3.1 Comparative proteomic results of osteoblastic cells versus osteosarcoma cells

In the present study, iTRAQ combined with NanoLC‐MS/MS analysis was used to determine differentially expressed proteins between osteoblastic cells (hFOB 1.19) and osteosarcoma cells (MNNG/HOS, MG63, and U2OS). After filtering for an unused protein score > 1.3 and peptide number ≥ 2, 4719 of 5290 proteins were identified and 4663 of 5218 proteins were quantified in the first and second runs, respectively. In total, we identified 5677 proteins and quantified 5601 proteins in the two replicates (Fig. 1B). Twenty‐five up‐regulated proteins and 41 down‐regulated proteins were identified and quantified in all osteosarcoma cell lines compared to the osteoblastic cell line (Fig. 1C,D). Table S1 lists the proteins that were up‐regulated and down‐regulated in osteosarcoma cells. GO analysis and KEGG pathway analysis were performed on the differentially expressed proteins (Fig. S1). Twenty‐five up‐regulated proteins and eight down‐regulated proteins were selected to further validate the proteomic results. The mRNA expression levels of these proteins observed in qRT‐PCR were consistent with the proteomic analysis (Fig. 1E–G). Then, nine up‐regulated proteins – FAS, CNOT1, ACACA, LRPPRC, FAK, ASNS, HMGCS1, PHGDH, and EFHD2 – and two down‐regulated proteins – MX1 and TAGLN – were chosen for protein expression verification. The expression levels of these proteins were consistent with the proteomic analysis (Fig. 1H). These results collectively support the feasibility of our approach for identifying osteosarcoma‐associated proteins.

### 3.2 The role of CNOT1 in osteosarcoma cells *in vitro*

**Figure 2:**
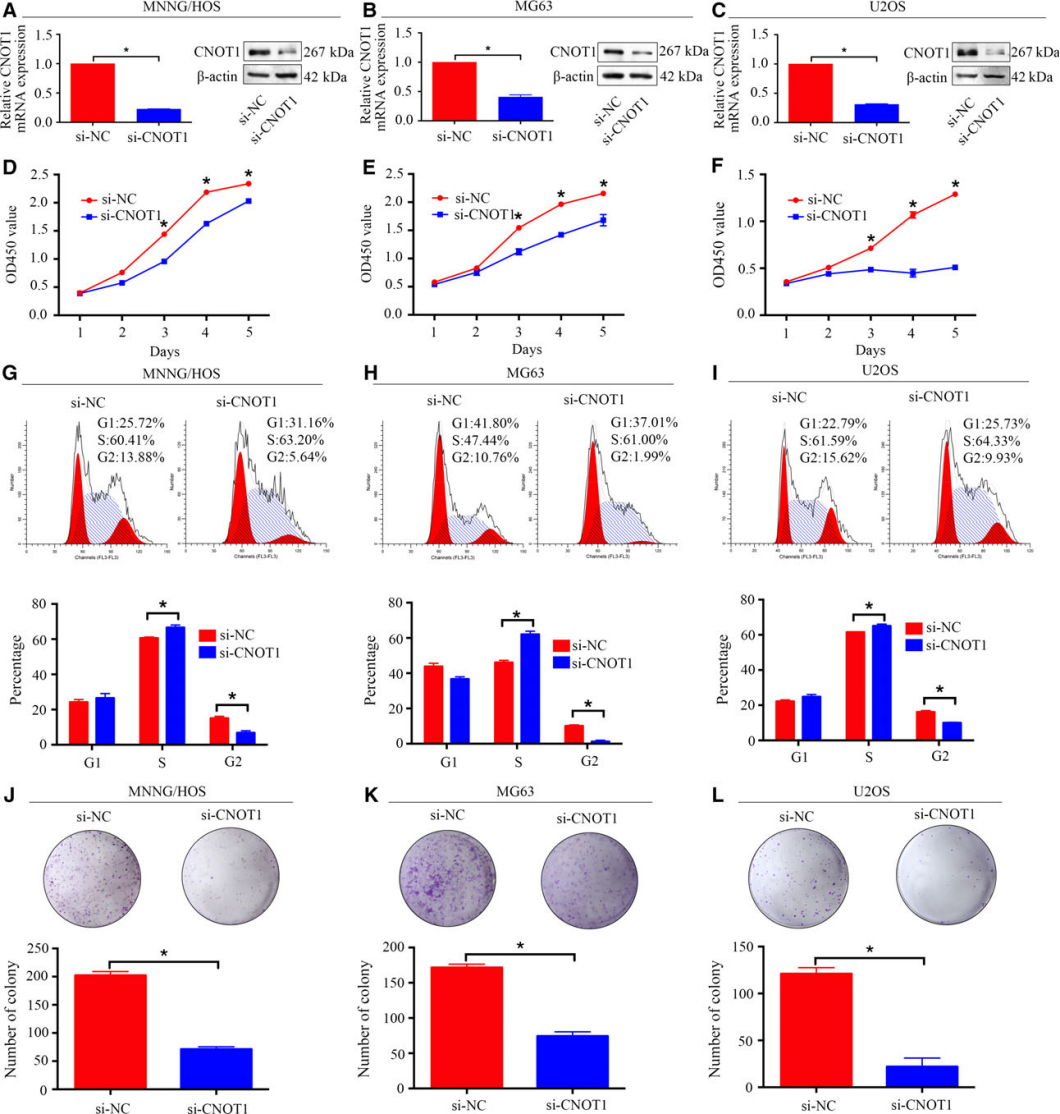
Knockdown of CNOT1 inhibits osteosarcoma cell proliferation *in vitro*. (A–C) The mRNA and protein expression levels were validated after si‐CNOT1 and si‐NC transfection by qRT‐PCR and western blotting in MNNG/HOS, MG63, and U2OS cells. (D–F) CCK‐8 assay was performed after siRNA transfection. (G–I) Cell cycle assays were performed for CNOT1‐silenced osteosarcoma cells and control cells. (J–L) colony forming assay for CNOT1‐silenced osteosarcoma cells and control cells. Data are representative of the results from three independent experiments. *P < 0.05.

Clinical analysis of nine up‐regulated proteins was conducted in 20 pairs of human tissue samples; each pair comprised an osteosarcoma sample and a corresponding nontumor tissue sample. The results revealed that the mRNA expression of CNOT1, ASNS, or EFHD2 was higher in osteosarcoma tissues than in the corresponding nontumor tissues (Fig. S2), whereas no difference was found for the six other proteins. siRNA knockdown and CCK‐8 assay were used for functional screening. The results showed that knockdown of CNOT1 dramatically inhibited osteosarcoma cell proliferation in MNNG/HOS cells (Fig. S3). These results showed that CNOT1 played an important role in the tumorigenesis of osteosarcoma and deserved further research. To explore the functional significance of CNOT1 in osteosarcoma, cell proliferation analysis, cell cycle assay, and colony forming assay were performed. CNOT1 knockdown was validated using qRT‐PCR and western blotting. The mRNA and protein expression of CNOT1 significantly decreased after transfection with CNOT1‐specific siRNA in osteosarcoma cells (Fig. 2A–C). CCK‐8 assay revealed that knockdown of CNOT1 significantly inhibited the proliferation of osteosarcoma cells (Fig. 2D–F). The cell cycle assay showed that knockdown of CNOT1 delayed cell cycle progression by inhibiting S phase transition (Fig. 2G–I). Similarly, colony forming assay showed that knockdown of CNOT1 attenuated cell colony formation (Fig. 2J–L). However, the migration and invasion assays showed that CNOT1 knockdown did not influence the migration or invasion of osteosarcoma, as shown in Fig. S4. Thus, these results indicate that CNOT1 has an oncogenic role in osteosarcoma.

### 3.3 Knockdown of CNOT1 inhibits tumor growth *in vivo*

**Figure 3:**
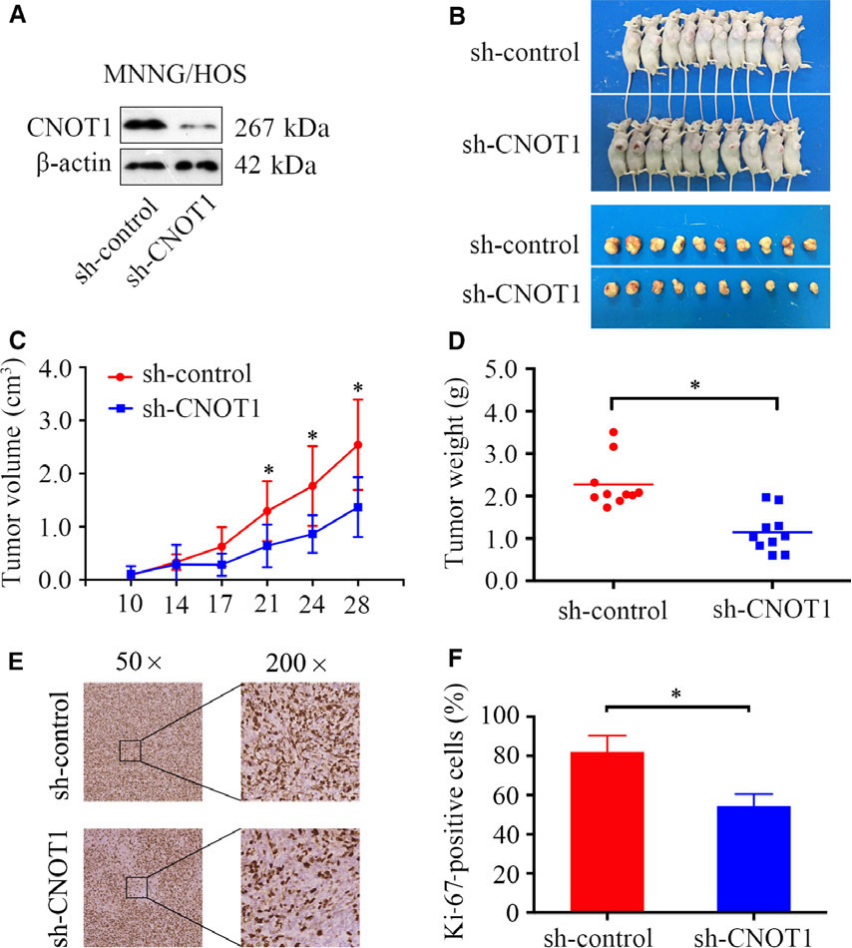
Knockdown of CNOT1 inhibits osteosarcoma cells growth *in vivo*. (A) Representative blots display the protein expression of CNOT1 in MNNG/HOS cells stably expressing sh‐control or sh‐CNOT1. β‐Actin was used as an internal control. (B) The upper diagram shows a photograph of tumor‐bearing mice, and the lower shows a photograph of tumors when the mice were euthanized. (C) Growth curve drawn by measuring tumor volumes on the indicated days. Error bars represent the SEM. (D) Diagram showing the tumor weights in the sh‐control group and sh‐CNOT1 group. (E,F) Representative images of Ki‐67 staining in the sh‐control group and sh‐CNOT1 group. Magnification, ×50, ×200.

To further determine the effect of CNOT1 on osteosarcoma growth *in vivo*, MNNG/HOS cells stably expressing sh‐control or sh‐CNOT1 were established. Protein expression was validated by western blotting (Fig. 3A). Then, the cells were subcutaneously injected into the left scapulas of nude mice, and the animals were closely monitored for tumor growth for 4 weeks. The tumor growth curve demonstrated that knockdown of CNOT1 significantly inhibited tumor growth *in vivo*. The tumor volume and weight were smaller in the CNOT1‐knockdown group than in the scramble group (Fig. 3B–D). The immunohistochemical analysis results shown in Fig. 3E and 3F indicated that the staining of the Ki‐67, a tissue proliferation marker, was lower in the sh‐CNOT1 tumors than in the sh‐control tumors (Fig. 3E,F). Taken together, these results indicated that CNOT1 knockdown hindered the tumorigenesis of osteosarcoma cells *in vivo*.

### 3.4 CNOT1 interacts with LMNA in osteosarcoma

**Figure 4:**
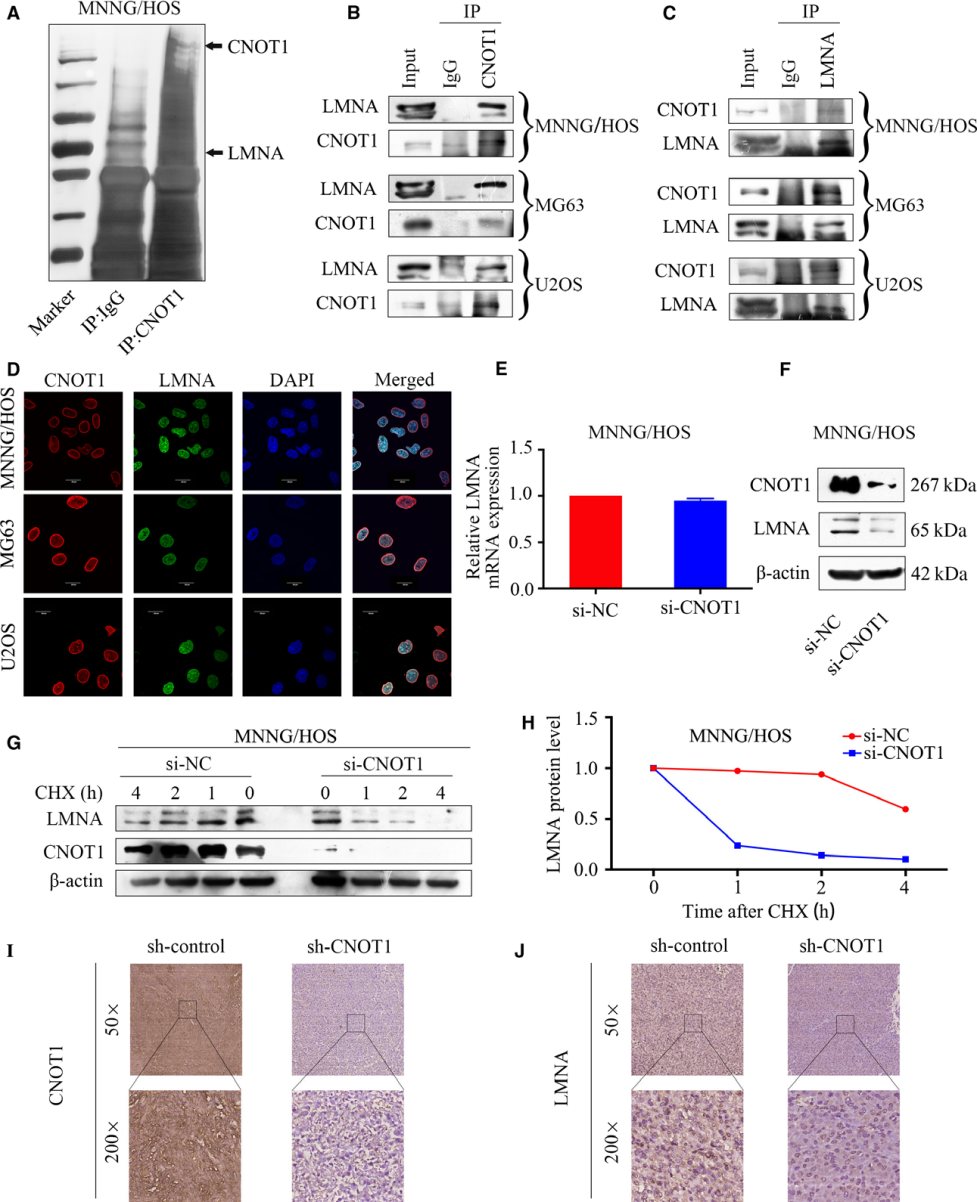
CNOT1 interacts with LMNA and affects its stability in osteosarcoma cells. (A) Silver staining of the SDS/PAGE after IgG or CNOT1 pulldown in MNNG/HOS cell line. (B) Whole‐cell lysates were immunoprecipitated with anti‐CNOT1 antibody followed by immunoblotting (IB) with anti‐LMNA and anti‐CNOT1 antibodies in the indicated osteosarcoma cell lines. IgG was used as a negative control. (C) Whole‐cell lysates were immunoprecipitated with anti‐LMNA antibody followed by IB with anti‐CNOT1 and anti‐LMNA antibodies in the indicated osteosarcoma cell lines. IgG was used as a negative control. (D) Immunofluorescence analysis performed using anti‐CNOT1 and anti‐LMNA antibodies. DAPI was used as a control for nuclear staining. (E,F) MNNG/HOS cells were transfected with si‐NC or si‐CNOT1 for 48 h. The effect of CNOT1 knockdown on LMNA mRNA and protein expression in MNNG/HOS cells was evaluated by qRT‐PCR and western blotting. β‐Actin was used as an internal control. (G,H) MNNG/HOS cells were transfected with si‐NC or si‐CNOT1 for 48 h, followed by treatment with cycloheximide (CHX; 100 mg·mL^−1^) for 0, 1, 2, and 4 h. Lysates were immunoblotted with anti‐LMNA or anti‐CNOT1. LMNA band intensity was normalized to β‐actin and then normalized to the time = 0 controls. (I) The expression of CNOT1 was detected in tumor tissue of sh‐control mice and sh‐CNOT1 mice by IHC. Magnification, × 50, × 200. (J) The expression of LMNA was detected in tumor tissue of sh‐control mice and sh‐CNOT1 mice by IHC. Magnification, × 50, × 200.

In order to determine the underlying mechanism of CNOT1 in osteosarcoma, Co‐IP followed by mass spectrometry identification was performed in MNNG/HOS cells. The silver staining of SDS/PAGE is shown in Fig. 4A. The identified proteins by mass spectrometry analysis (unused score > 1.3; peptides ≥ 2) are shown in Table S4. To assess the interaction between CNOT1 and other proteins, we pulled down CNOT1. The results showed that CNOT1 specifically interacted with LMNA in osteosarcoma cells as indicated by the Co‐IP assay (Fig. 4B). In addition, we also pulled down LMNA and probed with CNOT1 and found that LMNA interacted with CNOT1 (Fig. 4C). Similarly, we also confirmed the colocalization of LMNA and CNOT1 in osteosarcoma cells by confocal analysis (Fig. 4D). Thus, CNOT1 associates with LMNA in osteosarcoma cells. Next, we determined whether the mRNA and protein levels of LMNA were affected by CNOT1. Knockdown of CNOT1 in MNNG/HOS cells did not alter LMNA mRNA expression but led to a significant decrease in LMNA protein expression (Fig. 4E,F). To further clarify whether CNOT1 affects LMNA protein stability, we measured the LMNA protein half‐lives using cycloheximide (CHX) chase assay. The CHX chase assay results showed that knockdown of CNOT1 significantly decreased the LMNA protein half‐life (Fig. 4G,H). Then the expressions of CNOT1 and LMNA in the tumor tissues of tumor‐bearing mice were detected by IHC analysis. The results showed that CNOT1 expression was decreased in sh‐CNOT1 mice compared to the sh‐control mice (Fig. 4I). Similarly, the expression of LMNA was decreased in sh‐CNOT1 mice compared to sh‐NC mice (Fig. 4J). Taken together, these results strengthen the conclusion that CNOT1 interacts with LMNA and functions as a positive regulator of LMNA protein in osteosarcoma.

### 3.5 Knockdown of CNOT1 inhibits the Hedgehog signaling pathway in osteosarcoma

**Figure 5:**
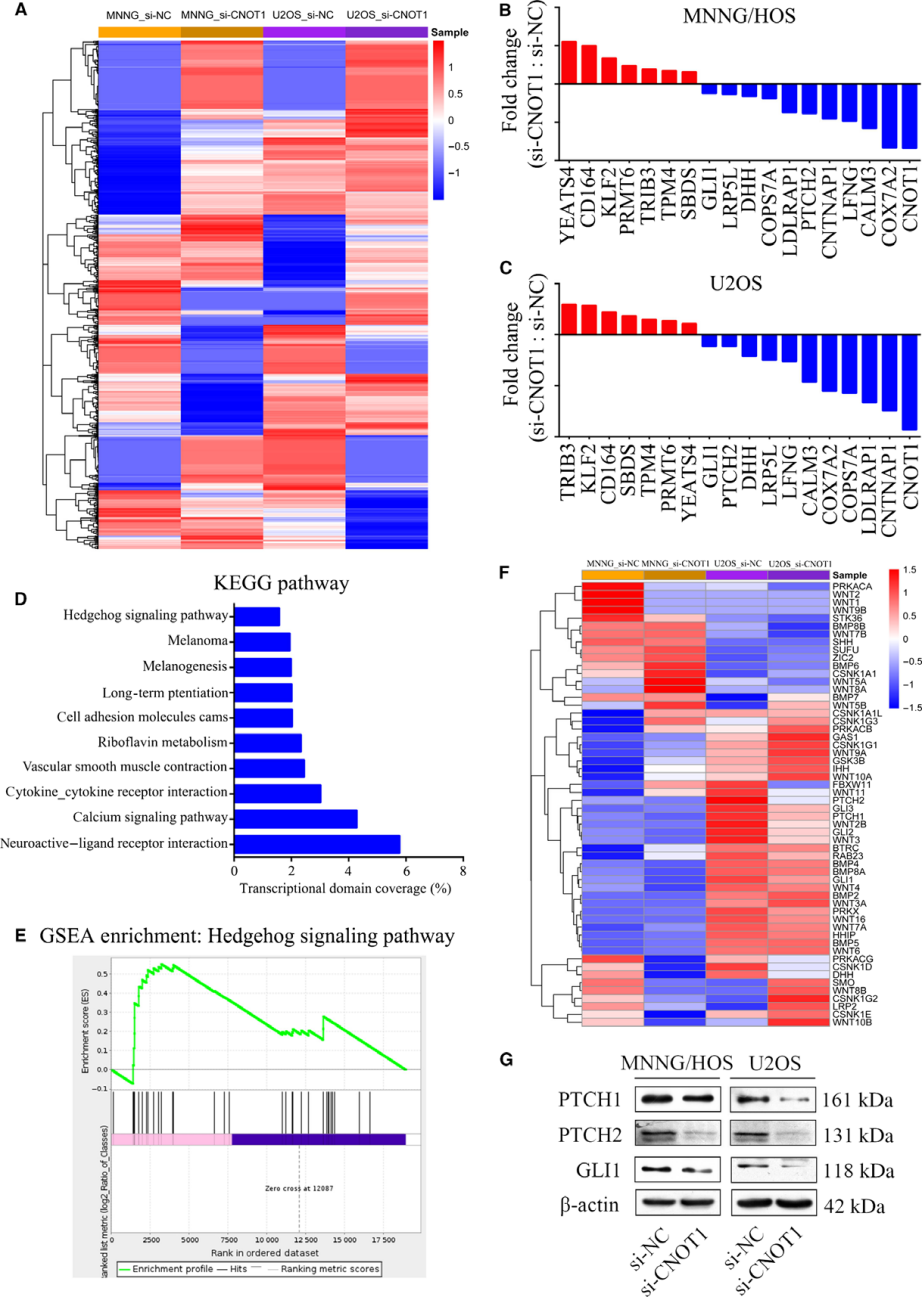
Knockdown of CNOT1 inhibits the Hedgehog signaling pathway in osteosarcoma cells. (A) Heat map of the genes that were differentially expressed between the si‐NC‐treated group and the si‐CNOT1‐treated group in MNNG/HOS and U2OS cells. (B,C) The mRNA expression results of seven up‐regulated genes and 11 down‐regulated genes were chosen for validation by qRT‐PCR. (D) The KEGG pathway analysis of the differentially expressed genes. (E) GSEA demonstrated an enrichment of gene signatures associated with the Hedgehog signaling pathway. (F) Heat map showing the change in genes enriched in the Hedgehog signaling pathway. (G) Representative blots showing the protein expression of GLI1, PTCH1, and PTCH2 in MNNG/HOS and U2OS cells after si‐CNOT1 transfection. β‐Actin was used as an internal control.

To further clarify the underlying mechanism of CNOT1, RNA‐seq analysis was performed to compare the global difference in gene expression between the si‐NC group and si‐CNOT1 group in MNNG/HOS cells and U2OS cells. The analysis revealed that 798 genes were commonly deregulated in MNNG/HOS and U2OS cells after transfection with si‐CNOT1 (Fig. 5A). Firstly, seven up‐regulated genes and 11 down‐regulated genes were chosen to verify the results of the analysis (Fig. 5B,C). The KEGG pathway analysis and Gene Set Enrichment analysis (GSEA) revealed an enrichment of the Hedgehog signaling pathway (Fig. 5D,E). Figure 5F shows the changes in the genes enriched in the Hedgehog signaling pathway. Then, three key proteins in the Hedgehog signaling pathway, including GLI1, PTCH1, and PTCH2, were chosen to detect the effect of CNOT1 on the pathway. The results showed that knockdown of CNOT1 significantly inhibited the expression of GLI1, PTCH1, and PTCH2 in osteosarcoma cells. Therefore, knockdown of CNOT1 inhibits the Hedgehog signaling pathway in osteosarcoma.

### 3.6 Knockdown of CNOT1 inhibits osteosarcoma cell proliferation through LMNA

**Figure 6:**
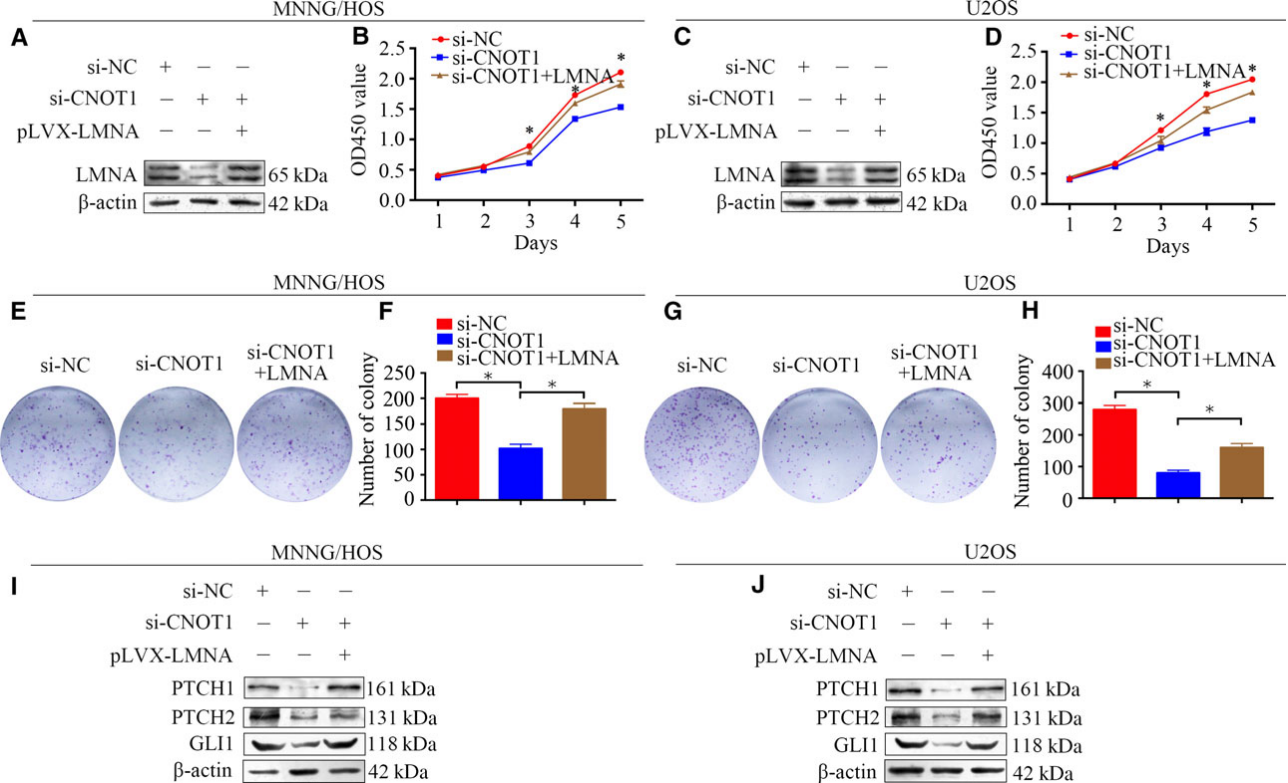
Knockdown of CNOT1 inhibits osteosarcoma cell proliferation through LMNA. (A,C) Representative blots of LMNA after transfection with pLVX‐Pruo‐Flag‐LMNA plasmid plus si‐CNOT1 or si‐NC. β‐Actin was used as an internal control. (B,D) CCK‐8 assay was used to detect tumor cell proliferation after transfection with pLVX‐Pruo‐Flag‐LMNA plasmid plus si‐CNOT1 or si‐NC. (E, F, G, H) colony forming assay was performed after transfection with pLVX‐Pruo‐Flag‐LMNA plasmid plus si‐CNOT1 or si‐NC. (I, J) Representative blots of GLI1, PTCH1, and PTCH2 after transfection with pLVX‐Pruo‐Flag‐LMNA plasmid plus si‐CNOT1 or si‐NC. β‐Actin was used as an internal control.

Our data demonstrated that CNOT1 interacted with LMNA and affected its protein stability in osteosarcoma cells. We hypothesized that CNOT1 functioned through LMNA. We overexpressed LMNA in CNOT1‐knockdown MNNG/HOS and U2OS cells. Western blotting confirmed that the LMNA protein level was up‐regulated in CNOT1‐knockdown cells after pLVX‐Pruo‐Flag‐LMNA plasmid transfection (Fig. 6A,C). As expected, the CCK‐8 assay and colony forming assay revealed that LMNA overexpression attenuated the CNOT1 knockdown‐induced cell growth inhibition (Fig. 6B,D,E–H). Then, we determined whether the change in the Hedgehog signaling pathway was attenuated in CNOT1‐knockdown cells after LMNA overexpression. The results showed that increasing the expression of LMNA in MNNG/HOS and U2OS cells reversed the down‐regulation of GLI1, PTCH1, and PTCH2 induced by CNOT1 knockdown (Fig. 6I,J). Taken together, knockdown of CNOT1 inhibits osteosarcoma cell proliferation by inhibition of the Hedgehog signaling pathway via LMNA.

### 3.7 CNOT1 is a prognostic marker for osteosarcoma

**Figure 7:**
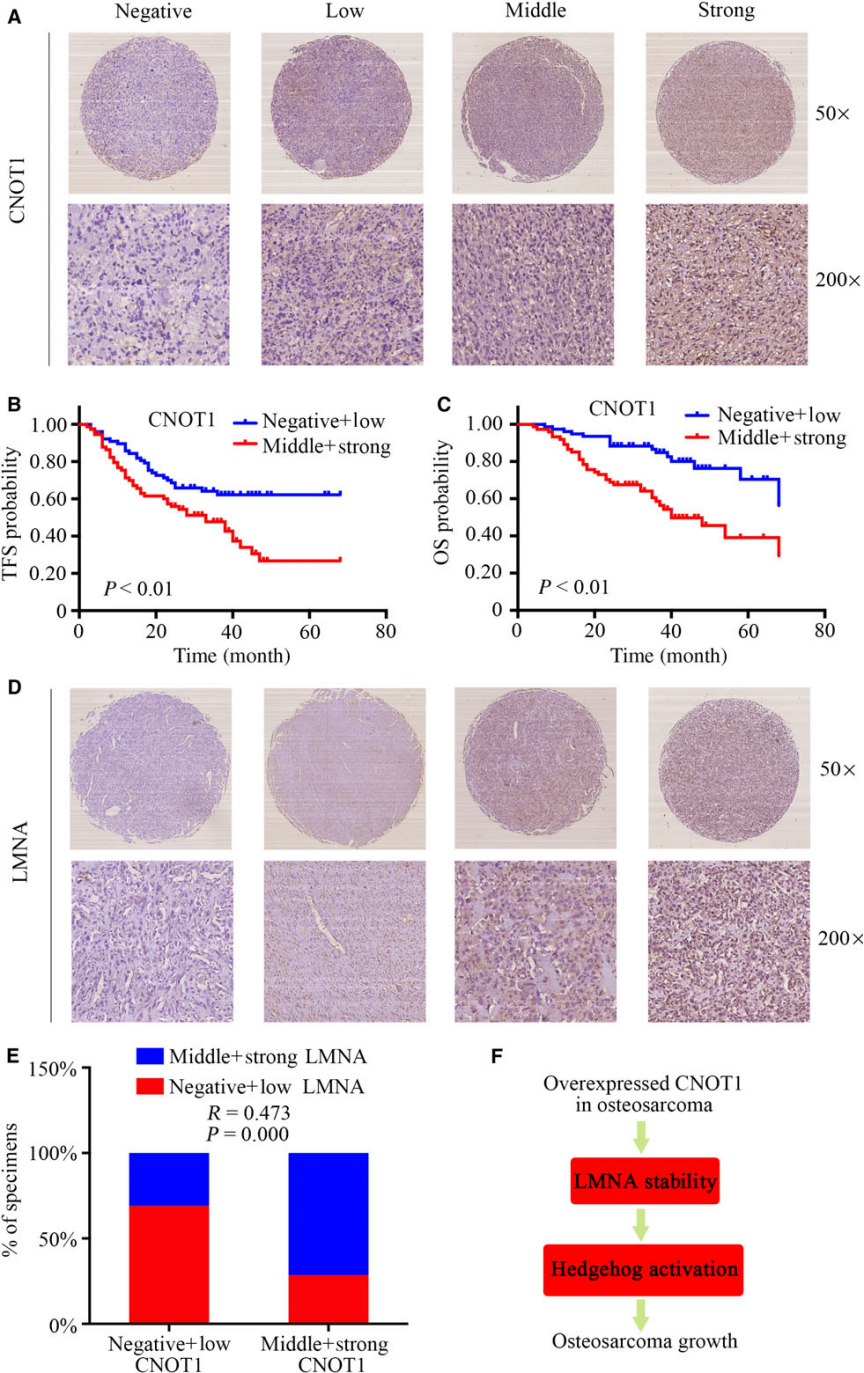
Clinical significance of CNOT1 in patients with osteosarcoma. (A) Representative IHC images of the expression levels of CNOT1 in osteosarcoma tissues. Original magnification: 50 ×, 200 ×. (B,C) The impact of CNOT1 level on TFS and OS of patients with osteosarcoma. (D) Representative IHC images of the expression levels of LMNA in osteosarcoma tissues. Original magnification: 50 ×, 200 ×. (E) Spearman's correlation analysis between CNOT1 protein levels and LMNA protein levels in 151 cases of osteosarcoma tissues. (F) Schematic representation of the function and potential mechanism of CNOT1 in osteosarcoma.

To further determine the clinicopathologic significance of CNOT1 in osteosarcoma, we performed IHC analysis of CNOT1 in a tissue microarray that included an independent set of 151 cases of osteosarcoma. Representative images of CNOT1 are shown in Fig. 7A. The correlations between the CNOT1 expression level and the clinicopathologic characteristics of the patients with osteosarcoma are summarized in Table 1. The expression level of CNOT1 was higher in patients with a clinically advanced Enneking stage than in those with an early stage (*P* = 0.000). Further analysis indicated that CNOT1 levels were correlated positively with recurrence (*P* = 0.005), indicating that CNOT1 expression played an important role in osteosarcoma recurrence. Univariate analysis showed that TFS was related to CNOT1 (*P* = 0.001) and Enneking stage (*P* = 0.001). OS was related to CNOT1 (*P* = 0.000) and Enneking stage (*P* = 0.000) (Table 2). Variables that were identified as significantly different in univariate analysis were used for multivariate analysis. The Cox proportional hazards model showed that CNOT1 (χ^2^ = 4.415, HR = 1.306, *P* = 0.036) and Enneking stage (χ^2^ = 3.867, HR = 1.722, *P* = 0.049) were independent prognostic variables for TFS. In addition, the Cox proportional hazards model showed that CNOT1 (χ^2^ = 5.518, HR = 1.425, *P* = 0.023) and Enneking stage (χ^2^ = 5.011, HR = 2.052, *P* = 0.025) were independent prognostic variables for OS (Table 3). The TFS and OS curves for CNOT1 are presented in Fig. 7B,C. The TFS and OS curves for Enneking stage are presented in Fig. S5. All other factors, including gender, age, tumor location, tumor necrosis rate, and cortical destruction, had no significant influence on prognosis. We also performed Kaplan–Meier survival analyses using microarray data (http://www.kmplot.com) from lung cancer and gastric cancer patients. We found that CNOT1 expression also correlated negatively with patient OS in lung cancer and gastric cancer (Fig. S6). Finally, we detected the expression of LMNA in osteosarcoma tissues. Representative images of LMNA are shown in Fig. 7D. A Spearman correlation analysis revealed a significant correlation between the expression levels of CNOT1 and LMNA (*R *=* *0.473, *P* = 0.000) in osteosarcoma tissues (Fig. 7E). In summary, CNOT1 was correlated with LMNA in tumor samples and associated with poor prognosis in patients with osteosarcoma.

**Table 1:** Correlation analyses of CNOT1 protein expression in relation to clinicopathologic variables of 151 patients with osteosarcoma

Clinicopathologic parameters	CNOT1 expression level				*P* value
	Negative	Low	Middle	Strong	
Gender					
Male	13	25	22	25	0.193
Female	18	21	15	12	
Age (years)					
6–20	16	24	20	21	0.071
20–40	13	10	9	14	
40–60	1	10	4	1	
60–84	1	2	4	1	
Location					
Femur	13	24	18	26	0.234
Tibia	10	14	11	9	
Elsewhere	8	8	8	2	
Tumor necrosis rate (%)					
< 90	23	38	30	30	0.824
≥90	8	8	7	7	
Cortical destruction					
Yes	24	42	30	31	0.380
No	7	4	7	6	
Recurrence					
Yes	9	19	18	26	0.005^a^
No	22	27	19	11	
Metastasis					
Yes	20	22	20	23	0.426
No	11	24	17	14	
Enneking stage					
II	28	43	26	16	0.000^a^
III	3	3	11	21	

a *P *<* *0.05.

**Table 2:** Impact of prognostic factors on TFS and OS by univariate analysis in osteosarcoma

Clinicopathologic parameters	Tumor‐free survival				Overall survival			
	NO.	HR	95% CI	*P*	NO.	HR	95% CI	*P*
Gender								
Male	81	0.780	0.489–1.244	0.297	81	0.586	0.333–1.032	0.064
Female	70				70			
Age (years)								
6–20	82	1.227	0.948–1.586	0.120	82	1.239	0.911–1.685	0.172
20–40	46				46			
40–60	16				16			
60–84	7				7			
Location								
Femur	81	0.983	0.724–1.334	0.912	81	0.859	0.585–1.262	0.439
Tibia	44				44			
Elsewhere	26				26			
Tumor necrosis rate (%)								
≥ 90	121	0.595	0.313–1.133	0.114	121	0.636	0.299–1.352	0.239
≥ 90	30				30			
Cortical destruction								
Yes	127	1.215	0.622–2.371	0.569	127	1.301	0.586–2.887	0.518
No	24				24			
CNOT1								
Negative	31	1.454	1.161–1.821	0.001^a^	31	1.676	1.277–2.199	0.000^a^
Low	46				46			
Middle	37				37			
Strong	37				37			
Enneking stage								
II	113	2.268	1.402–3.668	0.001^a^	113	2.985	1.726–5.161	0.000^a^
III	38				38			

*P *<* *0.05.

**Table 3:** Variables predictive of survival by COX proportional hazards model in osteosarcoma

	Parameters	Wald χ^2^	Risk Ratio	95% CI	*P*
TFS	CNOT1	4.415	1.306	1.018–1.675	0.036
	Enneking stage	3.867	1.722	1.002–2.960	0.049
OS	CNOT1	5.158	1.425	1.050–1.935	0.023
	Enneking stage	5.011	2.052	1.094–3.849	0.025

## 4 Discussion

The 5‐year survival rate for osteosarcoma has remained almost unchanged over the last three decades. Thus, there is an urgent need to explore new therapeutic targets. In this study, quantitative proteomics was used to compare the proteome profiles of osteosarcoma cells and human osteoblastic cells. Twenty‐five up‐regulated proteins and 41 down‐regulated were identified and quantified in osteosarcoma cells compared with osteoblastic cells. Then, 33 potential proteins associated with the tumorigenesis of osteosarcoma were selected for proteomic validation by qRT‐PCR, 11 of which were verified by western blotting. Many of the verified proteins have been reported to be closely associated with carcinogenesis. Zhou *et al*. (2014) reported that LRPPRC played a critical role in the development of prostate cancer and that its inhibition could present a potential molecular approach for the treatment of prostate cancer. Yu *et al*. (2016) demonstrated that knockdown of ASNS suppressed gastric cancer cell proliferation and inhibited tumor growth *in vivo*. FAK and PHGDH were reported to be novel prognostic biomarkers in gastric cancer (Du *et al*., 2014; Xian *et al*., 2016). In the present study, we detected the expression of nine verified up‐regulated proteins by qRT‐PCR in 20 pairs of osteosarcoma tissues and their corresponding nontumor tissues. The results showed that CNOT1, ASNS, and EFHD2 were overexpressed in osteosarcoma tissues compared with normal tissues, suggesting that they might play an important role in the tumorigenesis of osteosarcoma. Functional screening was conducted, and the results showed that knockdown of CNOT1 significantly inhibited osteosarcoma cell proliferation, suggesting an important role of CNOT1 in osteosarcoma carcinogenesis.

Currently, the role of CNOT1 in carcinogenesis remains unclear. In a previous study, CNOT1 was reported to act as a transcriptional regulator of nuclear receptor signaling by interacting with RXR in a ligand‐dependent manner (Winkler *et al*., 2006). Temme *et al*. (2010) investigated the role of CNOT1 in mRNA deadenylation. They found that CONT1 was required for bulk poly (A) shortening and hsp70 mRNA deadenylation. In addition, it was reported that CNOT1 depletion induced apoptosis by destroying the CCR4–NOT‐associated deadenylase activity. In the present study, we found that knockdown of CNOT1 resulted in significant cell growth inhibition in osteosarcoma cells *in vitro*. To further clarify the role of CNOT1 in osteosarcoma proliferation *in vivo*, MNNG/HOS cells with stable CNOT1 depletion were injected into nude mice. The results showed that CNOT1 knockdown could also dramatically impede tumor growth potential *in vivo*.

To identify the underlying mechanism of CNOT1, we screened CNOT1‐associated proteins using Co‐IP followed by mass spectrometry analysis. In the present study, we found that CNOT1 and LMNA interacted with each other in osteosarcoma cells. LMNA has been implicated in DNA damage response pathways by facilitating the rapid recruitment of 53BP1, a nucleoskeleton protein, to the sites of DNA damage (Gibbs‐Seymour *et al*., 2015). Depletion of LMNA enhances DNA damage‐induced replication fork arrest, indicating the requirement of LMNA in maintaining genomic stability (Singh *et al*., 2013). Willis *et al*. (2008) found that the expression of LMNA was strongly correlated with poor prognosis in colorectal cancer patients. This study, for the first time, demonstrates the interaction of CNOT1 with LMNA in osteosarcoma cells. Knockdown of CNOT1 affected the protein expression level of LMNA in osteosarcoma cells. To determine whether CNOT1 functioned through LMNA, a rescue experiment was performed. Restoration of the protein expression of LMNA rescued tumor cell growth, showing that the control of cell proliferation by CNOT1 was largely dependent on LMNA.

To further determine the underlying mechanism of CNOT1, RNA‐seq analysis was performed to identify the differentially expressed genes following si‐CNOT1 transfection. The KEGG pathway and GSEA analysis showed an enrichment of the Hedgehog signaling pathway. The Hedgehog signaling pathway has been shown to contribute to the growth and chemoresistance of osteosarcoma and other tumors (Hirotsu *et al*., 2010; Shahi *et al*., 2008, 2014). Emerging data suggest that interference with the Hedgehog signaling pathway by inhibitors may reduce osteosarcoma cell proliferation and tumor growth, thereby potentially improving the survival of patients with osteosarcoma (Kumar and Fuchs, 2015). We found that CNOT1 depletion inhibited the expression of GLI1, PTCH1, and PTCH2, which were key proteins in the Hedgehog signaling pathway. Then, we clarified the role of LMNA in CNOT1‐mediated Hedgehog signaling pathway regulation. The results showed that overexpression of LMNA rescued the inhibition of the Hedgehog signaling pathway induced by CNOT1 depletion.

## 5 Conclusions

Collectively, knockdown of CNOT1 dramatically inhibits osteosarcoma cell growth *in vitro* and *in vivo*. More importantly, this report provides, for the first time, experimental evidence and implicates the significance of the interaction between CNOT1 and LMNA in osteosarcoma cells. Knockdown of CNOT1 inhibits osteosarcoma cell proliferation by inhibition of the Hedgehog signaling pathway via LMNA, as shown in Fig. 6E. Notably, the expression of CNOT1 is an independent biomarker for TFS and OS in patients with osteosarcoma. Therefore, interventions targeting the CNOT1–LMNA–Hedgehog signaling pathway axis may be a feasible and effective strategy for the treatment of osteosarcoma.

## Author contributions

D‐DC and JL wrote the manuscript and designed this experiment. S‐JL helped to conduct the experiment. Q‐CY and C‐YF are in charge of the whole experiment conduction and paper writing. All of the authors reviewed the manuscript before submission and approved the final manuscript.

## Supporting information


**Fig. S1.** GO analysis and KEGG pathway analysis of the differentially expressed proteins between osteosarcoma cells and osteoblastic cells.

**Fig. S2.** The expression of these 9 up‐regulated proteins was measured using qRT‐PCR in 20 human tissue sample pairs in which each pair consisted of an osteosarcoma sample and a corresponding nontumor tissue sample.

**Fig. S3.** SiRNA knockdown and CCK‐8 assay was performed to search for osteosarcoma‐related protein in MNNG/HOS cell line.

**Fig. S4.** Transwell assays were used to detect the migration and invasion capability of each cell line after transfection with si‐CSE1L.

**Fig. S5.** The impact of Enneking stage on TFS and OS of patients with osteosarcoma.

**Fig. S6.** Kaplan–Meier survival analyses were performed using microarray data (http://www.kmplot.com) in lung cancer and gastric cancer patients.

**Table S1.** Lists of proteins that were up‐regulated and down‐regulated in osteosarcoma cells compared with osteoblast.

**Table S2.** The primer sequences for qRT‐PCR.

**Table S3.** The primary antibody for western blotting.

**Table S4.** The identified proteins by mass spectrometry after CNOT1 pulldown.
