# Supplementary material for: CNOT1 cooperates with LMNA to aggravate osteosarcoma tumorigenesis through the Hedgehog signaling pathway
Source: Mol Oncol. 2017 Mar 6;11(4):388–404. doi: 10.1002/1878-0261.12043 (PMC5527480; doi:10.1002/1878-0261.12043)
Supplement: Supplementary file 7 — Table S1. Lists of proteins that were up‐regulated and down‐regulated in osteosarcoma cells compared with osteoblast. [file MOL2-11-388-s007.docx]

Supplementary Table S1 Lists of proteins that were up-regulated and down-regulated in osteosarcoma cells compared with osteoblast.

| Accession number | Protein description | | Gene name | | 115:114 (mean±SD) | | 116:114 (mean±SD) | | 117:114 (mean±SD) | |  |
| --- | --- | --- | --- | --- | --- | --- | --- | --- | --- | --- | --- |
| sp\|P42704\|LPPRC | Leucine-rich PPR motif-containing protein, mitochondrial | | LRPPRC | | 8.77±0.86 | | 2.20±0.21 | | 4.41±0.49 | |  |
| sp\|P08243\|ASNS | Asparagine synthetase [glutamine-hydrolyzing] | | ASNS | | 4.63±2.62 | | 3.28±1.43 | | 12.47±3.84 | |  |
| sp\|P49327\|FAS | Fatty acid synthase | | FASN | | 7.45±2.01 | | 5.05±1.30 | | 2.29±0.68 | |  |
| sp\|O43175\|SERA | D-3-phosphoglycerate dehydrogenase | | PHGDH | | 5.01±1.35 | | 4.18±1.24 | | 8.24±2.05 | |  |
| sp\|P10809\|CH60 | 60 kDa heat shock protein, mitochondrial | | HSPD1 | | 5.60±0.29 | | 3.84±0.25 | | 3.16±0.04 | |  |
| sp\|Q05397\|FAK1 | Focal adhesion kinase 1 | | PTK2 | | 4.28±1.50 | | 6.55±4.36 | | 6.85±3.29 | |  |
| sp\|Q9Y6E2\|BZW2 | Basic leucine zipper and W2 domain-containing protein 2 | | BZW2 | | 3.74±1.54 | | 3.42±0.95 | | 2.56±0.29 | |  |
| sp\|P04406\|G3P | Glyceraldehyde-3-phosphate dehydrogenase | | GAPDH | | 5.07±0.53 | | 2.27±0.25 | | 7.45±0.05 | |  |
| sp\|Q96C19\|EFHD2 | EF-hand domain-containing protein D2 | | EFHD2 | | 4.79±0.37 | | 2.36±0.03 | | 2.00±0.52 | |  |
| sp\|P08195\|4F2 | 4F2 cell-surface antigen heavy chain | | SLC3A2 | | 3.98±0.16 | | 3.27±0.06 | | 3.80±0.14 | |  |
| sp\|P78527\|PRKDC | DNA-dependent protein kinase catalytic subunit | | PRKDC | | 5.02±1.67 | | 4.70±1.42 | | 2.83±1.29 | |  |
| sp\|P12956\|XRCC6 | X-ray repair cross-complementing protein 6 | | XRCC6 | | 3.41±0.22 | | 2.52±0.12 | | 2.17±0.36 | |  |
| sp\|Q13085\|ACACA | Acetyl-CoA carboxylase 1 | | ACACA | | 3.92±0.69 | | 8.00±2.31 | | 2.51±0.50 | |  |
| sp\|P07195\|LDHB | L-lactate dehydrogenase B chain | | LDHB | | 3.52±0.21 | | 3.48±0.02 | | 1.75±0.05 | |  |
| sp\|Q92616\|GCN1L | Translational activator GCN1 | | GCN1L1 | | 3.22±0.48 | | 3.25±0.17 | | 2.78±0.17 | |  |
| sp\|P46781\|RS9 | 40S ribosomal protein S9 | | RPS9 | | 4.43±2.58 | | 3.84±1.53 | | 1.96±0.67 | |  |
| sp\|P06744\|G6PI | Glucose-6-phosphate isomerase | | GPI | | 2.79±0.33 | | 4.27±0.08 | | 5.35±0.14 | |  |
| sp\|P52272\|HNRPM | Heterogeneous nuclear ribonucleoprotein M | | HNRNPM | | 2.38±0.12 | | 2.01±0.34 | | 2.15±0.15 | |  |
| sp\|P43246\|MSH2 | DNA mismatch repair protein Msh2 | | MSH2 | | 2.77±0.56 | | 2.89±0.51 | | 4.06±1.39 | |  |
| sp\|O15212\|PFD6 | Prefoldin subunit 6 | | PFDN6 | | 2.44±0.17 | | 1.82±0.18 | | 1.98±0.31 | |  |
| sp\|Q01581\|HMCS1 | Hydroxymethylglutaryl-CoA synthase, cytoplasmic | | HMGCS1 | | 2.15±0.20 | | 2.66±0.21 | | 2.51±0.02 | |  |
| sp\|P49736\|MCM2 | DNA replication licensing factor MCM2 | | MCM2 | | 2.00±0.00 | | 5.81±0.30 | | 3.94±0.45 | |  |
| sp\|P52701\|MSH6 | DNA mismatch repair protein Msh6 | | MSH6 | | 2.47±0.74 | | 2.35±0.75 | | 3.66±1.91 | |  |
| sp\|P29692\|EF1D | Elongation factor 1-delta | | EEF1D | | 2.80±1.27 | | 4.80±1.97 | | 2.42±1.58 | |  |
| sp\|A5YKK6\|CNOT1 | CCR4-NOT transcription complex subunit 1 | | CNOT1 | | 1.80±0.18 | | 2.28±0.10 | | 1.75±0.08 | |  |
| sp\|P61158\|ARP3 | | Actin-related protein 3 | | ACTR3 | | 0.53±0.04 | | 0.28±0.03 | | 0.36±0.13 | |
| sp\|Q9NSV4\|DIAP3 | | Protein diaphanous homolog 3 | | DIAPH3 | | 0.47±0.04 | | 0.48±0.10 | | 0.29±0.02 | |
| sp\|Q02224\|CENPE | | Centromere-associated protein E | | CENPE | | 0.52±0.04 | | 0.52±0.12 | | 0.53±0.08 | |
| sp\|P04083\|ANXA1 | | Annexin A1 | | ANXA1 | | 0.47±0.02 | | 0.43±0.04 | | 0.28±0.05 | |
| sp\|P12081\|SYHC | | Histidine--tRNA ligase, cytoplasmic | | HARS | | 0.45±0.03 | | 0.47±0.01 | | 0.19±0.06 | |
| sp\|Q5JSH3\|WDR44 | | WD repeat-containing protein 44 | | WDR44 | | 0.47±0.06 | | 0.29±0.03 | | 0.20±0.03 | |
| sp\|Q13439\|GOGA4 | | Golgin subfamily A member 4 | | GOLGA4 | | 0.38±0.03 | | 0.35±0.05 | | 0.53±0.06 | |
| sp\|P46821\|MAP1B | | Microtubule-associated protein 1B | | MAP1B | | 0.39±0.01 | | 0.21±0.05 | | 0.23±0.01 | |
| sp\|P35237\|SPB6 | | Serpin B6 | | SERPINB6 | | 0.44±0.11 | | 0.47±0.12 | | 0.42±0.04 | |
| sp\|Q9UHB6\|LIMA1 | | LIM domain and actin-binding protein 1 | | LIMA1 | | 0.32±0.05 | | 0.35±0.02 | | 0.05±0.02 | |
| sp\|P52732\|KIF11 | | Kinesin-like protein KIF11 | | KIF11 | | 0.39±0.05 | | 0.51±0.05 | | 0.41±0.02 | |
| sp\|P28838\|AMPL | | Cytosol aminopeptidase | | LAP3 | | 0.38±0.04 | | 0.31±0.01 | | 0.41±0.03 | |
| sp\|Q03001\|DYST | | Dystonin | | DST | | 0.30±0.00 | | 0.16±0.06 | | 0.38±0.13 | |
| sp\|Q9Y6A5\|TACC3 | | Transforming acidic coiled-coil-containing protein 3 | | TACC3 | | 0.32±0.04 | | 0.12±0.03 | | 0.42±0.02 | |
| sp\|P14635\|CCNB1 | | G2/mitotic-specific cyclin-B1 | | CCNB1 | | 0.34±0.07 | | 0.23±0.08 | | 0.34±0.02 | |
| sp\|O43663\|PRC1 | | Protein regulator of cytokinesis 1 | | PRC1 | | 0.22±0.09 | | 0.39±0.10 | | 0.15±0.05 | |
| sp\|O43396\|TXNL1 | | Thioredoxin-like protein 1 | | TXNL1 | | 0.27±0.00 | | 0.49±0.03 | | 0.43±0.08 | |
| sp\|P26038\|MOES | | Moesin | | MSN | | 0.21±0.08 | | 0.60±0.00 | | 0.35±0.03 | |
| sp\|P15924\|DESP | | Desmoplakin | | DSP | | 0.23±0.04 | | 0.19±0.01 | | 0.12±0.04 | |
| sp\|Q9Y3Z3\|SAMH1 | | Deoxynucleoside triphosphate triphosphohydrolase SAMHD1 | | SAMHD1 | | 0.27±0.05 | | 0.12±0.02 | | 0.15±0.09 | |
| sp\|P60660\|MYL6 | | Myosin light polypeptide 6 | | MYL6 | | 0.18±0.03 | | 0.24±0.05 | | 0.41±0.07 | |
| sp\|Q9NQW6\|ANLN | | Actin-binding protein anillin | | ANLN | | 0.15±0.03 | | 0.40±0.04 | | 0.18±0.01 | |
| sp\|P50452\|SPB8 | | Serpin B8 | | SERPINB8 | | 0.19±0.04 | | 0.16±0.09 | | 0.33±0.08 | |
| sp\|P84157\|MXRA7 | | Matrix-remodeling-associated protein 7 | | MXRA7 | | 0.23±0.10 | | 0.32±0.05 | | 0.13±0.02 | |
| sp\|O75330\|HMMR | | Hyaluronan mediated motility receptor | | HMMR | | 0.18±0.05 | | 0.23±0.12 | | 0.14±0.03 | |
| sp\|Q8WWK9\|CKAP2 | | Cytoskeleton-associated protein 2 | | CKAP2 | | 0.16±0.05 | | 0.28±0.10 | | 0.16±0.05 | |
| sp\|P42771\|CD2A1 | | Cyclin-dependent kinase inhibitor 2A, isoforms 1/2/3 | | CDKN2A | | 0.13±0.01 | | 0.11±0.03 | | 0.24±0.09 | |
| sp\|P50453\|SPB9 | | Serpin B9 | | SERPINB9 | | 0.11±0.01 | | 0.13±0.13 | | 0.19±0.04 | |
| sp\|Q9H6S3\|ES8L2 | | Epidermal growth factor receptor kinase substrate 8-like protein 2 | | EPS8L2 | | 0.12±0.04 | | 0.11±0.02 | | 0.36±0.15 | |
| sp\|Q6UVK1\|CSPG4 | | Chondroitin sulfate proteoglycan 4 | | CSPG4 | | 0.11±0.02 | | 0.26±0.02 | | 0.09±0.04 | |
| sp\|P78559\|MAP1A | | Microtubule-associated protein 1A | | MAP1A | | 0.08±0.01 | | 0.18±0.04 | | 0.19±0.07 | |
| sp\|Q96CV9\|OPTN | | Optineurin | | OPTN | | 0.08±0.01 | | 0.27±0.02 | | 0.33±0.09 | |
| sp\|Q9UJ70\|NAGK | | N-acetyl-D-glucosamine kinase | | NAGK | | 0.08±0.00 | | 0.41±0.07 | | 0.56±0.14 | |
| sp\|O00469\|PLOD2 | | Procollagen-lysine,2-oxoglutarate 5-dioxygenase 2 | | PLOD2 | | 0.08±0.01 | | 0.37±0.04 | | 0.28±0.02 | |
| sp\|Q05682\|CALD1 | | Caldesmon | | CALD1 | | 0.04±0.00 | | 0.27±0.00 | | 0.45±0.07 | |
| sp\|P08133\|ANXA6 | | Annexin A6 | | ANXA6 | | 0.04±0.00 | | 0.55±0.05 | | 0.17±0.01 | |
| sp\|P32455\|GBP1 | | Interferon-induced guanylate-binding protein 1 | | GBP1 | | 0.04±0.01 | | 0.56±0.04 | | 0.03±0.04 | |
| sp\|P05120\|PAI2 | | Plasminogen activator inhibitor 2 | | SERPINB2 | | 0.04±0.01 | | 0.10±0.01 | | 0.07±0.00 | |
| sp\|P08729\|K2C7 | | Keratin, type II cytoskeletal 7 | | KRT7 | | 0.03±0.01 | | 0.06±0.00 | | 0.04±0.00 | |
| sp\|P20591\|MX1 | | Interferon-induced GTP-binding protein Mx1 | | MX1 | | 0.03±0.00 | | 0.08±0.04 | | 0.04±0.01 | |
| sp\|Q01995\|TAGL | | Transgelin | | TAGLN | | 0.02±0.01 | | 0.03±0.00 | | 0.07±0.03 | |
