# Supplementary material for: CNOT1 cooperates with LMNA to aggravate osteosarcoma tumorigenesis through the Hedgehog signaling pathway
Source: Mol Oncol. 2017 Mar 6;11(4):388–404. doi: 10.1002/1878-0261.12043 (PMC5527480; doi:10.1002/1878-0261.12043)
Supplement: Supplementary file 8 — Table S2. The primer sequences for qRT‐PCR. [file MOL2-11-388-s008.docx]

Supplementary Table S2 The primer sequences for qRT-PCR

| Gene name |  | Sequence (5'->3') |
| --- | --- | --- |
| LRPPRC | Forward | GATTGCCTGCCGATTGAACC |
|  | Reverse | ATGTGGGTCTCCGCGAATG |
| ASNS | Forward | CTGCACGCCCTCTATGACAA |
|  | Reverse | GGAGTCCAAGCCCCCTGATA |
| FASN | Forward | TCGTGTTGACTTCTCGCTCC |
|  | Reverse | CCATCTCTCAAGACCACGGC |
| PHGDH | Forward | CTCTCACGGGGGTTGTGAAT |
|  | Reverse | CTCTTTCAGGAGGCCGACAA |
| HSPD1 | Forward | TGACCCAACAAAGGTTGTGAGA |
|  | Reverse | CACCCATTCCAGGGTCCTTC |
| PTK2 | Forward | CAGGGTCCGATTGGAAACCA |
|  | Reverse | CTGAAGCTTGACACCCTCGT |
| BZW2 | Forward | GGAGCTTTCCGACTTCCTCC |
|  | Reverse | AAGCACCACCTCCTTGATCG |
| GADPH | Forward | TCGGAGTCAACGGATTTGGT |
|  | Reverse | TTCCCGTTCTCAGCCTTGAC |
| EFHD2 | Forward | GATGTTCAAGCAGTATGATGCCG |
|  | Reverse | TTGCGGAAGATCAGGAGGAAC |
| SLC3A2 | Forward | ACCCCTGTTTTCAGCTACGG |
|  | Reverse | GGTCTTCACTCTGGCCCTTC |
| PRKDC | Forward | AGGACCTATAGCGTTGTGCC |
|  | Reverse | GGGATCACTCAGGTAAGCCG |
| XRCC6 | Forward | TCATGGCAACTCCAGAGCAG |
|  | Reverse | AACCTTGGGCAATGTCAGGT |
| ACACA | Forward | TAAGGCGCTGGTTTGTGGAA |
|  | Reverse | GACCAAGCTGCGGATTTGC |
| LDHB | Forward | ATTACCCAAACACCGCGTGA |
|  | Reverse | TCCACACAGCCACACTTGAG |
| GCN1L1 | Forward | ACGATGAGGACAACACTCGC |
|  | Reverse | ATCCAGTCAATGCCGGACAC |
| RPS9 | Forward | GAAGCGGAGCCAACATGC |
|  | Reverse | ATACTCGCCGATCAGCTTCAG |
| GPI | Forward | GCTGCCCTATGACCAGTACC |
|  | Reverse | TCTTGGTGCCTTGGTGGATG |
| HNRNPM | Forward | ATTCAACGAGTGCGGCCA |
|  | Reverse | TCGAACGTCAATCTCTCGGC |
| MSH2 | Forward | GATCTTGGCTTGGACCCTGG |
|  | Reverse | GGCATCCTGGGCTTCTTCAT |
| PFDN6 | Forward | CTTGTCTCGCACCCAGTAGG |
|  | Reverse | GGCCAGTTCCTCTTTCACGA |
| HMGCS1 | Forward | TAGTGGCCTGGAAGCCTTTG |
|  | Reverse | TGCTGAGGTGAGTACTGTGC |
| MCM2 | Forward | TCCGAGTGCTTTGTCTCCAC |
|  | Reverse | AGCAGCCTGAATACGCAACT |
| MSH6 | Forward | GTGTATCGCAGTGTTGGATGT |
|  | Reverse | TCGTAATGCAAGGATGGCGT |
| EEF1D | Forward | ACAGACCCAGCACGTATCTC |
|  | Reverse | CCAGCAGGATGGAGGACTTG |
| CNOT1 | Forward | GATTCAGGAGTGTGCGTTGC |
|  | Reverse | TGGGCAGCGAAACCTCTAAG |
| SMAHD1 | Forward | GTAAGACTGCCCCCAACAGA |
|  | Reverse | GCTATAACATCGCCATCCTGC |
| MXRA7 | Forward | CTTCTGCTCGCCTGGCTAC |
|  | Reverse | TCTGAACTCTCTGCTCCTCCT |
| SERPINB8 | Forward | CCTCGCCGTGAAAGAGTGAT |
|  | Reverse | GGCATGAGAAGGGAGCATGA |
| CSPG4 | Forward | CACAGAGGAACCCTGGATGG |
|  | Reverse | TTCAGCGAGAGGAGCACTTG |
| DSP | Forward | TGAGCTGGCAAAGGCATCTAA |
|  | Reverse | TTCACCCTGGTTTCTGCCTC |
| SERPINB9 | Forward | CGCTGGACTAGGTGGCAG |
|  | Reverse | AAAGACAGTGCCTGGGCCAT |
| TAGLN | Forward | TAGGGAAACCCACCCTCTCA |
|  | Reverse | CTGGGGAAAGCTCCTTGGAA |
| MX1 | Forward | GCCTTCCGATTCCCCATTCA |
|  | Reverse | TGGACTTAGGTGCCTTGTGG |
| COX7A2 | Forward | CTCTTCGGCCCGGAAAGATT |
|  | Reverse | ACGAAGAGCCAGCAGATTCC |
| CALM3 | Forward | CAGCTGACTGAGGAGCAGAT |
|  | Reverse | TCAATGGTCCCGTTCCCATC |
| COPS7A | Forward | ACAATCCAGTCCCTCAGCCT |
|  | Reverse | TCAGCCAGCTCTCTAACATTGG |
| LFNG | Forward | CGCCACAAGGAGATGACGTT |
|  | Reverse | ATACTCCACGGCCATCTTGC |
| CNTNAP1 | Forward | CTTGGAGCCCAAGCCAGAAC |
|  | Reverse | CTCGTCGCAGCCGTAGTAG |
| LRP5L | Forward | CAGCCTTTCTGAGCCAAGGA |
|  | Reverse | ACCTCGTCATCCGTCCAGTA |
| LDLRAP1 | Forward | CCCCTCCTTGAAGAGCTTGG |
|  | Reverse | CCATCATCCAGCTCCCAGAC |
| TPM4 | Forward | AAGAAAGCCGCTGAGGACAA |
|  | Reverse | CATCACCTTCAGCGTCGGAG |
| TRIB3 | Forward | CGCGGCCAGATGCGA |
|  | Reverse | CGGGACGCTCGGTATCTAAG |
| SBDS | Forward | GCTGCCTGAAGCTAGTGAGT |
|  | Reverse | CATCGAGGTCTTTTTCCACGC |
| PRMT6 | Forward | AAGATGTCGCAGCCCAAGAA |
|  | Reverse | GTCCAGTACCGTCTTGCCTC |
| CD164 | Forward | GACTTTAGCGCCCATCTCCA |
|  | Reverse | TGTGGGTTTAGCGGAACAGA |
| YEATS4 | Forward | GCGGGAGAGTAAAGGGTGTT |
|  | Reverse | ATGATACAGGGTTACCTCATTTCT |
| KLF2 | Forward | ACTCACACCTGCAGCTACGC |
|  | Reverse | AGTGGTAGGGCTTCTCACCTGT |
| DHH | Forward | GTGCCGCTACTCTACAAGCA |
|  | Reverse | TACAACGCTCGGTCATCAGG |
| GLI1 | Forward | CCCGGAGTGCAGTCAAGTT |
|  | Reverse | CCAGAGATGGGCTCATGGTG |
| PTCH2 | Forward | GGCTTAGGCCCGAGGAGAT |
|  | Reverse | GGGACCCGAAGACCAATTCA |
| LMNA | Forward | GAGATGATCCCTTGCTGACTT |
|  | Reverse | CAGGTGTTCTGTGCCTTCCA |
