# Supplementary material for: CNOT1 cooperates with LMNA to aggravate osteosarcoma tumorigenesis through the Hedgehog signaling pathway
Source: Mol Oncol. 2017 Mar 6;11(4):388–404. doi: 10.1002/1878-0261.12043 (PMC5527480; doi:10.1002/1878-0261.12043)
Supplement: Supplementary file 9 — Table S3. The primary antibody for western blotting. [file MOL2-11-388-s009.docx]

Supplementary Table S3 The primary antibody for Western blotting

| Protein name | Dilution ratio | Reagent brand |
| --- | --- | --- |
| FAS | 1:500 | Bioworld Technology |
| CNOT1 | 1:500 | Proteintech |
| ACACA | 1:500 | Bioworld Technology |
| LRPPRC | 1:1000 | Proteintech |
| FAK | 1:500 | Bioworld Technology |
| ASNS | 1:500 | Bioworld Technology |
| HMGCS1 | 1:1000 | Proteintech |
| PHGDH | 1:100 | ABGENT |
| EFHD2 | 1:500 | Proteintech |
| MX1 | 1:500 | Bioworld Technology |
| TAGLN | 1:500 | Bioworld Technology |
| LMNA | 1:1000 | Proteintech |
| PTCH1 | 1:500 | BBI Life Sciences |
| PTCH2 | 1:200 | Proteintech |
| GLI1 | 1:250 | BBI Life Sciences |
| β-actin | 1:20000 | Sigma-Aldrich |
