# Supplementary material for: CNOT1 cooperates with LMNA to aggravate osteosarcoma tumorigenesis through the Hedgehog signaling pathway
Source: Mol Oncol. 2017 Mar 6;11(4):388–404. doi: 10.1002/1878-0261.12043 (PMC5527480; doi:10.1002/1878-0261.12043)
Supplement: Supplementary file 10 — Table S4. The identified proteins by mass spectrometry after CNOT1 pulldown. [file MOL2-11-388-s010.docx]

Supplementary Table S4 The identified proteins by mass spectrometry after CNOT1 pulldown.

| Accession # | Unused | Peptides(95%) |
| --- | --- | --- |
| sp\|P23246\|SFPQ_HUMAN | 52.93 | 58 |
| sp\|A5YKK6\|CNOT1_HUMAN | 78.94 | 45 |
| sp\|Q15233\|NONO_HUMAN | 37.15 | 44 |
| tr\|E9PB61\|E9PB61_HUMAN | 25.62 | 38 |
| sp\|P16403\|H12_HUMAN | 29.26 | 34 |
| sp\|P10412\|H14_HUMAN | 2.02 | 33 |
| sp\|P16402\|H13_HUMAN | 2 | 28 |
| sp\|P48634\|PRC2A_HUMAN | 46.88 | 25 |
| sp\|P04264\|K2C1_HUMAN | 34.57 | 23 |
| sp\|P62701\|RS4X_HUMAN | 27.86 | 22 |
| sp\|Q9UHX1-6\|PUF60_HUMAN | 29.52 | 19 |
| sp\|Q00839\|HNRPU_HUMAN | 26.74 | 19 |
| sp\|Q14498-2\|RBM39_HUMAN | 24.81 | 19 |
| tr\|A8MXA8\|A8MXA8_HUMAN | 17.92 | 18 |
| sp\|P13645\|K1C10_HUMAN | 31.53 | 16 |
| sp\|P35527\|K1C9_HUMAN | 18.76 | 12 |
| sp\|P61247\|RS3A_HUMAN | 16.57 | 12 |
| sp\|Q9UJV9\|DDX41_HUMAN | 24.4 | 11 |
| sp\|P49756\|RBM25_HUMAN | 20.2 | 11 |
| tr\|E9PKE3\|E9PKE3_HUMAN | 18.43 | 11 |
| sp\|P62081\|RS7_HUMAN | 14.43 | 11 |
| tr\|Q6UYC3\|Q6UYC3_HUMAN | 16.82 | 10 |
| sp\|P35908\|K22E_HUMAN | 14.48 | 10 |
| sp\|P26368\|U2AF2_HUMAN | 11.08 | 10 |
| sp\|Q9BY77\|PDIP3_HUMAN | 18.94 | 9 |
| sp\|O00541\|PESC_HUMAN | 16.33 | 9 |
| sp\|Q9Y383\|LC7L2_HUMAN | 16.09 | 9 |
| sp\|Q9UNQ2\|DIM1_HUMAN | 13.36 | 9 |
| tr\|F8VYX6\|F8VYX6_HUMAN | 15.98 | 8 |
| sp\|Q9BVI4\|NOC4L_HUMAN | 14.15 | 8 |
| tr\|F5H5D3\|F5H5D3_HUMAN | 13.58 | 8 |
| sp\|Q92522\|H1X_HUMAN | 11.23 | 8 |
| sp\|P42766\|RL35_HUMAN | 4.22 | 8 |
| tr\|B7Z1E5\|B7Z1E5_HUMAN | 15.17 | 7 |
| sp\|Q7L014\|DDX46_HUMAN | 13.19 | 7 |
| sp\|Q9NVP1\|DDX18_HUMAN | 13.15 | 7 |
| sp\|P32969\|RL9_HUMAN | 11.92 | 7 |
| sp\|P26373\|RL13_HUMAN | 9.51 | 7 |
| sp\|P01857\|IGHG1_HUMAN | 8.45 | 7 |
| sp\|P08107\|HSP71_HUMAN | 3.57 | 7 |
| sp\|Q9C0C2\|TB182_HUMAN | 12.49 | 6 |
| sp\|Q9BYG3\|MK67I_HUMAN | 12.29 | 6 |
| sp\|Q8WXX5\|DNJC9_HUMAN | 11.97 | 6 |
| sp\|P11387\|TOP1_HUMAN | 11.56 | 6 |
| sp\|O95232\|LC7L3_HUMAN | 11.52 | 6 |
| sp\|Q5JTH9\|RRP12_HUMAN | 11.42 | 6 |
| sp\|P62280\|RS11_HUMAN | 10.88 | 6 |
| tr\|E7EU87\|E7EU87_HUMAN | 1.55 | 6 |
| sp\|Q9UKZ1\|CB029_HUMAN | 9.55 | 5 |
| sp\|Q9BVP2\|GNL3_HUMAN | 9.12 | 5 |
| sp\|P15880\|RS2_HUMAN | 8.29 | 5 |
| sp\|Q9H6F5\|CCD86_HUMAN | 7.6 | 5 |
| sp\|P67809\|YBOX1_HUMAN | 7.29 | 5 |
| sp\|Q9BZE4\|NOG1_HUMAN | 7.18 | 5 |
| sp\|Q96EY4\|CD043_HUMAN | 6.82 | 5 |
| tr\|E7ETL9\|E7ETL9_HUMAN | 4.5 | 5 |
| tr\|Q6IRZ0\|Q6IRZ0_HUMAN | 4.03 | 5 |
| sp\|O75533\|SF3B1_HUMAN | 8.49 | 4 |
| sp\|P62277\|RS13_HUMAN | 8.4 | 4 |
| sp\|P18621\|RL17_HUMAN | 8.25 | 4 |
| sp\|Q9NV06\|DCA13_HUMAN | 8.02 | 4 |
| tr\|F5H0N0\|F5H0N0_HUMAN | 7.41 | 4 |
| sp\|Q9BQG0\|MBB1A_HUMAN | 7.3 | 4 |
| sp\|Q9NUL7\|DDX28_HUMAN | 7.11 | 4 |
| sp\|Q5VTL8\|PR38B_HUMAN | 6.9 | 4 |
| tr\|E7EVA0\|E7EVA0_HUMAN | 6.35 | 4 |
| tr\|E5RIT6\|E5RIT6_HUMAN | 6.27 | 4 |
| tr\|Q8IWE6\|Q8IWE6_HUMAN | 6.1 | 4 |
| sp\|Q8NFW8\|NEUA_HUMAN | 5.92 | 4 |
| sp\|Q01081\|U2AF1_HUMAN | 5.68 | 4 |
| sp\|Q6DKI1\|RL7L_HUMAN | 5.56 | 4 |
| tr\|C9JNW5\|C9JNW5_HUMAN | 4.45 | 4 |
| sp\|Q9BTM1\|H2AJ_HUMAN | 4.01 | 4 |
| sp\|P62753\|RS6_HUMAN | 3.9 | 4 |
| sp\|Q969Q0\|RL36L_HUMAN | 3.29 | 4 |
| tr\|E7ESU4\|E7ESU4_HUMAN | 2.92 | 4 |
| sp\|P09661\|RU2A_HUMAN | 2.44 | 4 |
| sp\|Q9NQ29\|LUC7L_HUMAN | 0.59 | 4 |
| tr\|E7EUB4\|E7EUB4_HUMAN | 7.47 | 3 |
| sp\|P62424\|RL7A_HUMAN | 6.85 | 3 |
| sp\|Q9BQ67\|GRWD1_HUMAN | 6.75 | 3 |
| tr\|F5H012\|F5H012_HUMAN | 6.4 | 3 |
| sp\|Q9P258\|RCC2_HUMAN | 6.21 | 3 |
| sp\|Q9BX40\|LS14B_HUMAN | 6.21 | 3 |
| tr\|E9PHK9\|E9PHK9_HUMAN | 6.01 | 3 |
| sp\|Q66PJ3\|AR6P4_HUMAN | 6 | 3 |
| sp\|Q8NC51\|PAIRB_HUMAN | 5.93 | 3 |
| tr\|B3KRJ9\|B3KRJ9_HUMAN | 5.92 | 3 |
| sp\|P62807\|H2B1C_HUMAN | 5.82 | 3 |
| tr\|G3V108\|G3V108_HUMAN | 5.8 | 3 |
| tr\|F8WJN3\|F8WJN3_HUMAN | 5.64 | 3 |
| sp\|P63173\|RL38_HUMAN | 5.6 | 3 |
| tr\|G3V203\|G3V203_HUMAN | 5.49 | 3 |
| sp\|O75175\|CNOT3_HUMAN | 5.4 | 3 |
| tr\|F8W9L2\|F8W9L2_HUMAN | 5.15 | 3 |
| sp\|Q9UMS4\|PRP19_HUMAN | 5.07 | 3 |
| sp\|Q86W92\|LIPB1_HUMAN | 4.86 | 3 |
| sp\|Q9Y3Y2\|CHTOP_HUMAN | 4.15 | 3 |
| sp\|Q9UMY1\|NOL7_HUMAN | 4.11 | 3 |
| sp\|Q9Y295\|DRG1_HUMAN | 4.07 | 3 |
| tr\|Q5JR95\|Q5JR95_HUMAN | 3.38 | 3 |
| sp\|P62829\|RL23_HUMAN | 5.31 | 2 |
| tr\|F8WAF2\|F8WAF2_HUMAN | 4.92 | 2 |
| tr\|E9PD90\|E9PD90_HUMAN | 4.83 | 2 |
| tr\|E7EWF1\|E7EWF1_HUMAN | 4.82 | 2 |
| sp\|P82933\|RT09_HUMAN | 4.68 | 2 |
| tr\|E5RIL2\|E5RIL2_HUMAN | 4.4 | 2 |
| sp\|P05141\|ADT2_HUMAN | 4.35 | 2 |
| tr\|E9PC97\|E9PC97_HUMAN | 4.33 | 2 |
| sp\|P62854\|RS26_HUMAN | 4.33 | 2 |
| sp\|Q5SSJ5\|HP1B3_HUMAN | 4.21 | 2 |
| tr\|E9PIZ3\|E9PIZ3_HUMAN | 4 | 2 |
| sp\|P62913\|RL11_HUMAN | 4 | 2 |
| sp\|Q9Y3A4\|RRP7A_HUMAN | 3.92 | 2 |
| tr\|Q5T6W5\|Q5T6W5_HUMAN | 3.82 | 2 |
| tr\|G5E927\|G5E927_HUMAN | 3.67 | 2 |
| sp\|P21127\|CD11B_HUMAN | 3.63 | 2 |
| tr\|E9PHS3\|E9PHS3_HUMAN | 3.63 | 2 |
| sp\|Q9HCM4\|E41L5_HUMAN | 3.6 | 2 |
| sp\|P62266\|RS23_HUMAN | 3.6 | 2 |
| sp\|Q9UGN5\|PARP2_HUMAN | 3.58 | 2 |
| sp\|P62805\|H4_HUMAN | 3.57 | 2 |
| sp\|P46781\|RS9_HUMAN | 3.49 | 2 |
| sp\|Q6P087\|RUSD3_HUMAN | 3.43 | 2 |
| sp\|Q15459\|SF3A1_HUMAN | 3.39 | 2 |
| sp\|Q8IY81\|RRMJ3_HUMAN | 3.27 | 2 |
| sp\|P0CW22\|RS17L_HUMAN | 3.25 | 2 |
| tr\|E9PR30\|E9PR30_HUMAN | 3.23 | 2 |
| tr\|E9PBF6\|E9PBF6_HUMAN | 3.21 | 2 |
| sp\|P78316\|NOP14_HUMAN | 3.18 | 2 |
| sp\|Q9UNF1\|MAGD2_HUMAN | 3.07 | 2 |
| sp\|Q6PK04\|CC137_HUMAN | 3.05 | 2 |
| sp\|Q7Z417\|NUFP2_HUMAN | 3.05 | 2 |
| sp\|P84098\|RL19_HUMAN | 3.02 | 2 |
| sp\|O43290\|SNUT1_HUMAN | 2.86 | 2 |
| sp\|Q15393\|SF3B3_HUMAN | 2.8 | 2 |
| tr\|Q5J9I4\|Q5J9I4_HUMAN | 2.78 | 2 |
| tr\|E7EQ40\|E7EQ40_HUMAN | 2.72 | 2 |
| tr\|B7Z6S8\|B7Z6S8_HUMAN | 2.7 | 2 |
| sp\|Q9BU76\|MMTA2_HUMAN | 2.6 | 2 |
| sp\|Q9BW19\|KIFC1_HUMAN | 2.53 | 2 |
| sp\|Q8ND56\|LS14A_HUMAN | 2.48 | 2 |
| sp\|Q8N9M1\|CS047_HUMAN | 2.28 | 2 |
| tr\|F8VYE8\|F8VYE8_HUMAN | 2.16 | 2 |
| tr\|F8W9B5\|F8W9B5_HUMAN | 1.94 | 2 |
| tr\|Q6IPX4\|Q6IPX4_HUMAN | 1.93 | 2 |
| tr\|E9PG73\|E9PG73_HUMAN | 1.86 | 2 |
| tr\|E7EX53\|E7EX53_HUMAN | 1.76 | 2 |
| sp\|P20719\|HXA5_HUMAN | 1.71 | 2 |
| tr\|F2Z3A5\|F2Z3A5_HUMAN | 1.65 | 2 |
| sp\|Q7Z7K6\|CENPV_HUMAN | 1.45 | 2 |
| tr\|E9PPJ0\|E9PPJ0_HUMAN | 1.37 | 2 |
